# Pineal melatonin is a circadian time-giver for leptin rhythm in *Syrian hamsters*

**DOI:** 10.3389/fnins.2015.00190

**Published:** 2015-05-27

**Authors:** Ibtissam Chakir, Stéphanie Dumont, Paul Pévet, Ali Ouarour, Etienne Challet, Patrick Vuillez

**Affiliations:** ^1^Laboratory of Biology and Health, Faculty of Science, Abdelmalek Essaâdi UniversityTetouan, Morocco; ^2^Regulation of Circadian Clocks Team, Institute for Cellular and Integrative Neurosciences, UPR3212, Centre National de la Recherche Scientifique and University of StrasbourgStrasbourg, France

**Keywords:** golden hamster, pinealectomy, photoperiod, cortisol rhythm, plasma glucose

## Abstract

Nocturnal secretion of melatonin from the pineal gland may affect central and peripheral timing, in addition to its well-known involvement in the control of seasonal physiology. The Syrian hamster is a photoperiodic species, which displays gonadal atrophy and increased adiposity when adapted to short (winter-like) photoperiods. Here we investigated whether pineal melatonin secreted at night can impact daily rhythmicity of metabolic hormones and glucose in that seasonal species. For that purpose, daily variations of plasma leptin, cortisol, insulin and glucose were analyzed in pinealectomized hamsters, as compared to sham-operated controls kept under very long (16 h light/08 h dark) or short photoperiods (08 h light/16 h dark). Daily rhythms of leptin under both long and short photoperiods were blunted by pinealectomy. Furthermore, the phase of cortisol rhythm under a short photoperiod was advanced by 5.6 h after pinealectomy. Neither plasma insulin, nor blood glucose displays robust daily rhythmicity, even in sham-operated hamsters. Pinealectomy, however, totally reversed the decreased levels of insulin under short days and the photoperiodic variations in mean levels of blood glucose (i.e., reduction and increase in long and short days, respectively). Together, these findings in *Syrian hamsters* show that circulating melatonin at night drives the daily rhythmicity of plasma leptin, participates in the phase control of cortisol rhythm and modulates glucose homeostasis according to photoperiod-dependent metabolic state.

## Introduction

Photoperiodic animals, such as *Syrian hamsters*, are species whose physiology is specifically and reversibly regulated on a seasonal basis. Notably, they display seasonal changes in gonadal activity. When *Syrian hamsters* are exposed to long, summer-like photoperiods, gonadal function is active and their adiposity is decreased. Conversely, after transfer to short, winter-like photoperiods, these hamsters become sexually quiescent, while their adiposity increases (Bartness and Wade, 1985).

In mammals, melatonin can be synthesized by several organs, including the pineal gland, retina and gastrointestinal tract (Pevet, 2003; Hardeland et al., 2011; Tosini et al., 2012). The pineal gland, however, is the main source of the nocturnal peak of circulating melatonin, as evidenced by the lack of detectable levels in the blood after pinealectomy. The daily fluctuations of melatonin synthesis and release by the pineal are tightly controlled by the master clock in the suprachiasmatic nuclei of the hypothalamus (SCN). The daily duration of nocturnal melatonin transduces photoperiodic cues into neuroendocrine changes that modulate seasonal physiology, thus highlighting the pivotal role of pineal melatonin in the integration of seasonal changes in day length (Malpaux et al., 2001; Pevet, 2003).

Besides, the nocturnal peak of melatonin may also play a role as an internal time-giver on a daily basis (Pevet and Challet, 2011). Among others, circadian rhythmicity of the pups during gestation and weaning can be synchronized by maternal melatonin via placenta and milk (Torres-Farfan et al., 2008, 2011). In adults, nocturnal melatonin can control the rhythmic activity of brain and peripheral regions, including striatum, pars tuberalis of the hypophysis and spleen, as shown by the disappearance of clock gene oscillations in these structures after pinealectomy (Messager et al., 2001; Uz et al., 2003; Prendergast et al., 2013). Melatoninergic cues may even feed-back on the master clock in the SCN where they affect clock gene expression (Agez et al., 2007) and firing rate (Rusak and Yu, 1993).

The aim of the present study was to investigate whether rhythmic melatonin can also affect the daily timing of other hormones, such as leptin and glucocorticoids. Plasma leptin is secreted by adipocytes in proportion with adiposity (Ahima and Flier, 2000). Accordingly, levels of plasma leptin are higher in *Syrian hamsters* adapted to short photoperiod compared to animals exposed to long photoperiod (Horton et al., 2000). In addition, leptin is rhythmically secreted, with peak phases depending on the species. Adrenal glucorticoids are other hormones rhythmically secreted, with endogenous peaks occurring around activity onsets (Dickmeis, 2009). The main glucocorticoid is cortisol in humans and hamsters, and corticosterone in rats and mice. To test whether nocturnal melatonin can have chronomodulatory effects on leptin, cortisol and insulin rhythms, we investigated the impact of pinealectomy on these rhythms in *Syrian hamsters* kept under long or short photoperiods.

## Material and methods

### Animals

Ninety-seven male *Syrian hamsters (Mesocricetus auratus*) bred in-house (Chronobiotron platform, UMS3415, CNRS and University of Strasbourg) were 6-month-old at the end of the experiment. From birth, they were maintained in a Long Photoperiod (LP) consisting of 16-h light and 8-h dark (around 150 lux within the cages during the light period), with lights on at 05:00 AM, defining Zeitgeber time (ZT) 0. Animals were housed 3–5 per cage and kept at 22 ± 1°C with *ad libitum* access to water and food. All experiments were conducted in accordance with the French National Law (License 67–88) implementing the European Union Directive 2010/63/EU. All efforts were made to minimize the number of animals used and their suffering, and the study met the ethical standards.

### Experimental design

All hamsters were initially kept under LP. Around half of the animals were sham-operated (*n* = 48), while the others were pinealectomized (*n* = 49).

Hamsters (weighing approximately 140 g) were anesthetized during the light phase with i.p. injections of a mixture of Zoletil 20 (Virbac, Carros, France) and Rompun (Bayer Pharma, Puteaux, France). After being placed into a stereotaxic instrument (Kopf), a midline circular incision of the skull was gently performed to expose the pineal gland. The pineal was removed with a pair of fine forceps, the skull cap replaced and the incision closed. After surgery, hamsters were housed individually for 1–2 days until complete recovery. Thereafter, hamsters were either kept in LP (*n* = 21) or transferred to a Short Photoperiod (SP; *n* = 28) consisting of 8 h light and 16 h dark, with lights on at 09:00 AM, defining ZT0 under SP. This photoperiodic condition triggers in that species an inhibition of the reproductive axis, including gonadal atrophy.

Ten weeks later, all animals under LP and SP were killed every 4 h (*n* = 1 of the same cage per ZT) throughout the 24 h, from ZT3, 7, 11, 15, 19 to 23.

Blood samples were collected in heparinized tubes and centrifuged for 10 min at 4600 g at 4°C.

### Hormonal assays

Plasma concentrations of leptin were determined by a multi-species leptin RIA kit (XL-85K, Millipore, Molsheim, France). The limit of sensitivity of the assay was 2 ng·mL^−1^ and the inter- and intra-assay coefficients of variance were <9 and <4%, respectively.

Plasma concentrations of cortisol were determined by a Cortisol Express EIA Kit (AYN830, Cayman Chemical, Ann Arbor, MI, USA). The limit of sensitivity of the assay was 0.1 ng·mL^−1^, while the coefficients of variance for a dose of 5 ng were equal, respectively, to 11 and 6% for inter- and intra-assays. Plasma levels of insulin were assayed with Ultra Rat Insulin ELISA Kit (# 90060, Crystal Chem, Downers Grove, IL, USA) using hamster insulin standard (# 90330, Crystal Chem) following the instructions of the manufacturer. The limit of sensitivity of the assay was 0.1 ng·mL^−1^ and the coefficients of variance were <10% for both inter- and intra-assays.

### Glucose assay

Blood glucose was determined with GOD-PAP Kit (LP80009, Biolabo, Maizy, France).

### Statistical analysis

Data are presented as means ± S.E.M. Three-Way analyses of variance (ANOVA) followed by *post-hoc* comparisons with the Fisher's least significant difference Test were used to compare the effects of Zeitgeber time × photoperiod × treatment. Level of significance was set at *P* < 0.05. For each blood parameter in either photoperiod, One-Way ANOVAs, or ANOVAs on ranks when normality test (Shapiro–Wilk) failed, were performed to determine if the effect of Zeitgeber time was significant (i.e., if the blood parameters display time-dependent variations). For assessing daily rhythmicity, we used a cosinor analysis to determine mean level, amplitude and acrophase of the considered parameter with SigmaPlot software (Systat software Inc., San Jose, CA, USA). Individual data of each experimental group (Long or short photoperiod, sham-operated or pinealectomized) were fitted to the following regression: [y = a+b·cos(2·π·(x−c)/24)] where a is the mean level, b the amplitude, and c the acrophase of the rhythm. For a given parameter, we used the Mean, Size, standard Error (MSE) format of SigmaPlot to compare the acrophases of significant regressions with *t*-tests or One-Way ANOVA followed by Fisher's *post-hoc* test for two or more groups, respectively.

## Results

Body mass at the end of the experiment was not significantly modified by pinealectomy, either in LP or SP (Table 1). Pinealectomy is routinely used in this and other labs to suppress the nocturnal rise of circulating melatonin. Indeed, after visual extirpation of the pineal gland in hamsters, levels of plasma melatonin at night become systematically undetectable (i.e., less than 1 pg/tube according to the limit of sensitivity of our RIA melatonin assay) (Schuster et al., 2001). In this study, we attribute the differences in pinealectomized hamsters compared to sham-operated controls, as due to impaired melatonin secretion. Based on the present protocol without hormonal replacement in pinealectomized animals, we cannot fully exclude the possibility of melatonin-independent effects of pinealectomy.

**Table 1:** **Body mass of hamsters**.

	**LP Sham (*n* = 23)**	**LP PinX (*n* = 21)**	**SP Sham (*n* = 25)**	**SP PinX (*n* = 28)**
Mean (g)	129.7	127.6	136.5	131.9
±SEM	±2.9	±2.6	±2.7	±2.1

*Mean body mass of hamsters, either sham-operated (Sham) or pinealectomized (PinX) under long (LP) or short photoperiods (SP)*.

### Cortisol rhythm

**Figure 1:**
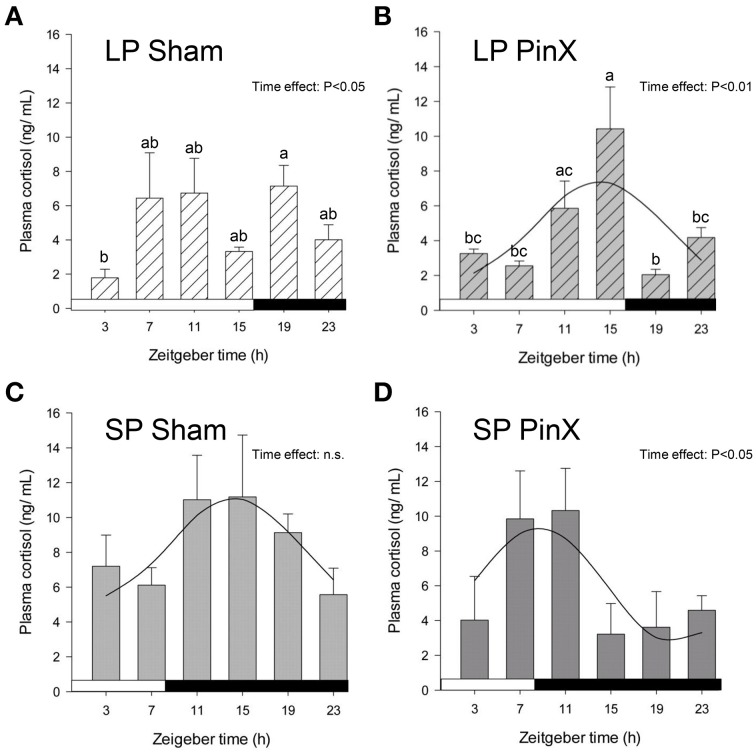
**Effects of photoperiod and pinealectomy on daily variations of plasma cortisol in the** ***Syrian hamster*****.** Light and dark bars represent photoperiod and scotoperiod, respectively. LP, long photoperiod (16 h light/08 h dark) **(A,B)**; SP, short photoperiod (08 h light/16 h dark) **(C,D)**; Sham, sham-operated hamsters **(A,C)**; PinX, pinealectomized hamsters **(B,D)**. Fitted curves represent significant cosinor analyses (for details, see Materials and Methods and Table 2). The absence of a curve in the LP Sham panel indicates that the daily variations of cortisol are not significant in these conditions. The inset for each graph indicates the effect of Zeitgeber time, evaluated by One-Way ANOVAs or ANOVAs on ranks when normality test (Shapiro-Wilk) failed; n.s., non-significant. For a given panel, histograms lacking common letters are significantly different (*P* < 0.05).

Levels of plasma cortisol were significantly affected by Zeitgeber time (Three-Way ANOVA; *P* = 0.01). Independently of photoperiod and treatment, higher values were found at ZT11 (i.e., 11 h after lights on) compared to ZT3 (early morning) and ZT23 (late night; Figure 1). There was also a significant effect of photoperiod (Three-Way ANOVA; *P* = 0.003) on plasma cortisol, mean values being larger under short days compared to long days. By contrast, plasma cortisol was not modified by Treatment (Three-Way ANOVA; sham-operated vs. pinealectomized; *P* = 0.09). In addition, the triple interaction [Zeitgeber time × photoperiod × treatment] was significant (Three-Way ANOVA; *P* = 0.004), but only for sham-operated animals, indicating that in these animals the daily pattern differs between photoperiods. Cosinor analysis detected a significant daily rhythm for both sham-operated and pinealectomized hamsters under short photoperiod (Table 2). Of note, the cortisol acrophase was phase-advanced by almost 6 h in pinealectomized hamsters compared to intact animals (from ZT14.3 to ZT8.7; *P* < 0.05; One-Way ANOVA; Figure 1; Table 2). By contrast, under long photoperiod, a significant daily rhythm was found only in pinealectomized hamsters (delayed by more than 5 h as compared to pinealectomized hamsters under short days; *P* < 0.05; One-Way ANOVA; Table 2). The lack of significant daily rhythmicity in intact animals under long days is probably due to the bimodal pattern characterized by daytime and nocturnal peaks (Figure 1).

**Table 2:** **Parameters of cosinor regressions**.

		**Plasma leptin**			**Plasma cortisol**			**Plasma insulin**			**Plasma glucose**		
		**Mean**	**SEM**	***P***	**Mean**	**SEM**	***P***	**Mean**	**SEM**	***P***	**Mean**	**SEM**	***P***
LP Sham (*n* = 23)	a	7.06	0.49	<0.001	5.03	0.71	<0.001	1.79	0.22	<0.001	1.02	0.05	<0.001
	b	2.18	0. 70	0.006	1.46	1.02	0.17	0.21	0.32	0.53	0.04	0.08	0.62
	c	21.34^*^	1.22	<0.001	13.43	2.60	<0.001	6.68	5.36	0.27	14.60	7.20	<0.001
LP PinX (*n* = 21)	a	6.95	0.67	<0.001	4.72	0.65	<0.001	1.78	0.10	<0.001	1.24	0.03	<0.001
	b	0.75	0.95	0.43	2.66	0.92	0.009	0.38	0.15	0.017	0.05	0.04	0.19
	c	6.75	4.80	0.17	14.08^*^	1.32	<0.001	4.84^*^	1.48	0.004	13.84	2.81	<0.001
SP Sham (*n* = 25)	a	8.48	0.61	<0.001	8.27	0.74	<0.001	1.32	0.09	<0.001	1.12	0.05	<0.001
	b	2.45	0.85	0.01	2.82	1.09	0.015	0.12	0.13	0.37	0.11	0.06	0.09
	c	13.22^#^	1.34	<0.001	14.26^*^	1.36	<0.001	18.92	4.22	0.11	18.80	2.36	<0.001
SP PinX (*n* = 28)	a	8.70	0.05	<0.001	6.01	0.88	<0.001	1.81	0.20	<0.001	1.00	0.035	<0.001
	b	0.11	0.06	0.91	3.30	1.21	0.011	0.61	0.28	0.037	0.10	0.05	0.041
	c	8.81	2.36	0.81	8.67^#^	1.45	<0.001	20.18^#^	1.79	<0.001	12.96	1.81	<0.001

The table shows the three parameters of cosinor regression, including a the mean level, b the amplitude, and c the acrophase of the rhythm (see Material and Methods for details). For the acrophase, the reference time is Zeitgeber 0 (i.e., lights on) for both photoperiods. LP, long photoperiod; SP, short photoperiod; Sham, sham-operated; PinX, pinealectomized. Italicized parameters are not statistically significant (P > 0.05). For a given hormone (column), mean acrophases lacking common superscripts (

* or

# *) are significantly different (t-test or One-Way ANOVA for 2 or more groups, respectively). No superscript is included in case the overall regression was not significant due to non-significant acrophase (c) and/or amplitude (b), or when there was only one significant regression precluding statistical comparison with the other groups (as in the case of plasma glucose)*.

### Leptin rhythm

**Figure 2:**
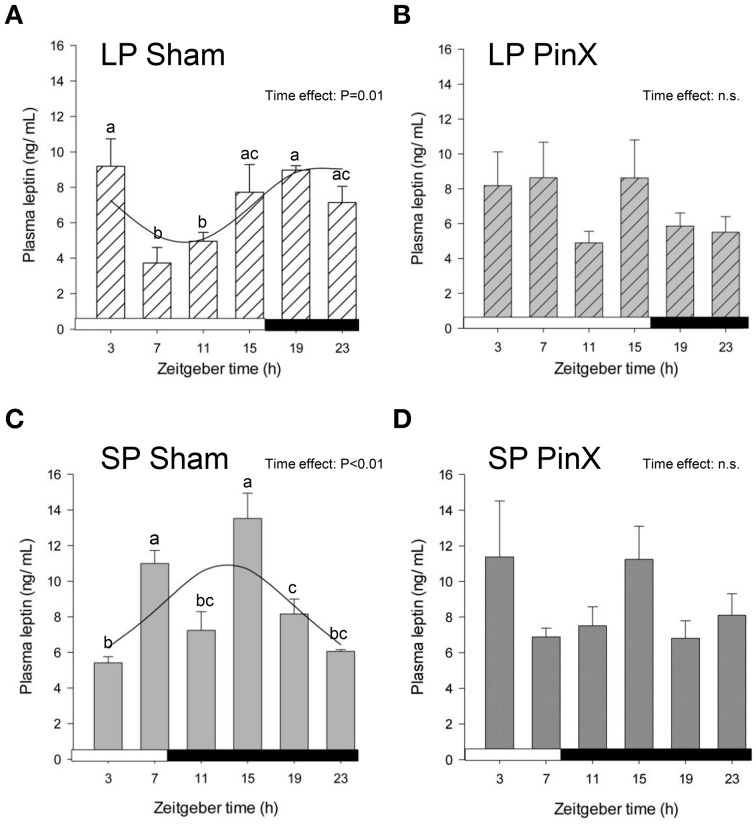
**Effects of photoperiod and pinealectomy on daily variations of plasma leptin in the** ***Syrian hamster*****.** Light and dark bars represent photoperiod and scotoperiod, respectively. LP, long photoperiod (16 h light/08 h dark) **(A,B)**; SP, short photoperiod (08 h light/16 h dark) **(C,D)**; Sham, sham-operated hamsters **(A,C)**; PinX, pinealectomized hamsters **(B,D)**. Fitted curves represent significant cosinor analyses (for details, see Materials and Methods and Table 2). The absence of a curve in LP PinX and SP PinX **(B,D)** indicates that the daily rhythmicity of leptin is not significant after pinealectomy. The inset for each graph indicates the effect of Zeitgeber time, evaluated by One-Way ANOVAs or ANOVAs on ranks when normality test (Shapiro-Wilk) failed; n.s., non-significant. For a given panel, histograms lacking common letters are significantly different (*P* < 0.05).

Levels of plasma leptin were changed according to Zeitgeber times (Three-Way ANOVA; *P* = 0.002), the values at ZT15 being larger than those at both ZT11 and ZT23 (Figure 2). Moreover, the levels of leptin were modified by photoperiod (Three-Way ANOVA; *P* = 0.004), with increased values in short days. Levels of plasma leptin were not affected by pinealectomy (Three-Way ANOVA; *P* = 0.9). However, the triple interaction [Zeitgeber time × photoperiod × treatment] was significant (Three-Way ANOVA; *P* = 0.003), but only after pinealectomy, indicating that the daily patterns of leptin in these animals differ in both photoperiods, as compared to respective controls (Figure 2). Accordingly, a significant rhythm was detected only in sham-operated animals, demonstrating a role of time-giver of circulating melatonin upon leptin rhythm (Figure 2; Table 2). Furthermore, the leptin acrophase under long photoperiod was delayed by 8 h as compared to short photoperiod (from ZT13.2 to ZT21.3; *P* < 0.05; *t*-test; Table 2).

### Insulin rhythm

**Figure 3:**
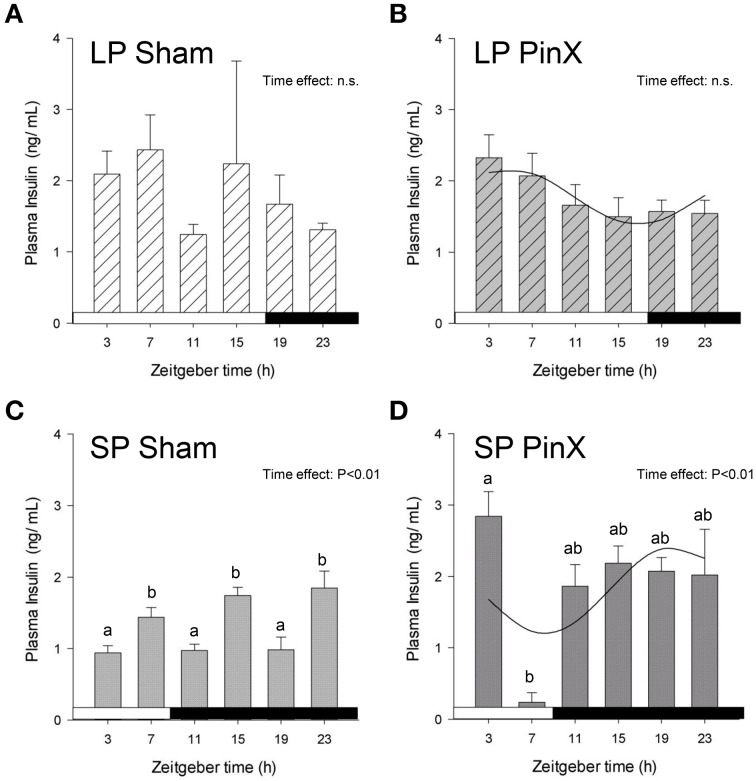
**Effects of photoperiod and pinealectomy on daily variations of plasma insulin in the** ***Syrian hamster*****.** Light and dark bars represent photoperiod and scotoperiod, respectively. LP, long photoperiod (16 h light/08 h dark) **(A,B)**; SP, short photoperiod (08 h light/16 h dark) **(C,D)**; Sham, sham-operated hamsters **(A,C)**; PinX, pinealectomized hamsters **(B,D)**. Fitted curves represent significant cosinor analyses (for details, see Materials and Methods and Table 2). The absence of a curve in LP Sham and SP Sham **(A,C)** indicates that the daily rhythmicity of insulin is not significantly rhythmic in sham-operated hamsters. The inset for each graph indicates the effect of Zeitgeber time, evaluated by One-Way ANOVAs or ANOVAs on ranks when normality test (Shapiro-Wilk) failed; n.s., non-significant. For a given panel, histograms lacking common letters are significantly different (*P* < 0.05).

The mains effects of Zeitgeber time, photoperiod and treatment were not significant for insulin levels (Three-Way ANOVA; *P* = 0.14, *P* = 0.17, and *P* = 0.11, respectively). However, the double interactions [Zeitgeber time × photoperiod] and [Zeitgeber time × treatment] were significant (Three-Way ANOVA; *P* = 0.01 for both), indicating that the daily profiles of insulin differ according to photoperiodic conditions. In particular, the mean levels of plasma insulin were lower in hamsters exposed to short photoperiod as compared to long photoperiod, but only for sham-operated animals (Fisher's *post-hoc* test; *P* = 0.02). Finally, the double interaction [photoperiod × treatment] was also significant (Three-Way ANOVA; *P* = 0.04), revealing that insulin levels were increased by pinealectomy under short photoperiod (Fisher's *post-hoc* test; *P* < 0.01), but they were unaffected by this treatment under long photoperiod (Figure 3; Table 2). Cosinor analysis detected a significant daily rhythm of plasma insulin only in pineactomized hamsters, with different acrophases between short and long photoperiods (Figure 3; Table 2).

### Glucose rhythm

**Figure 4:**
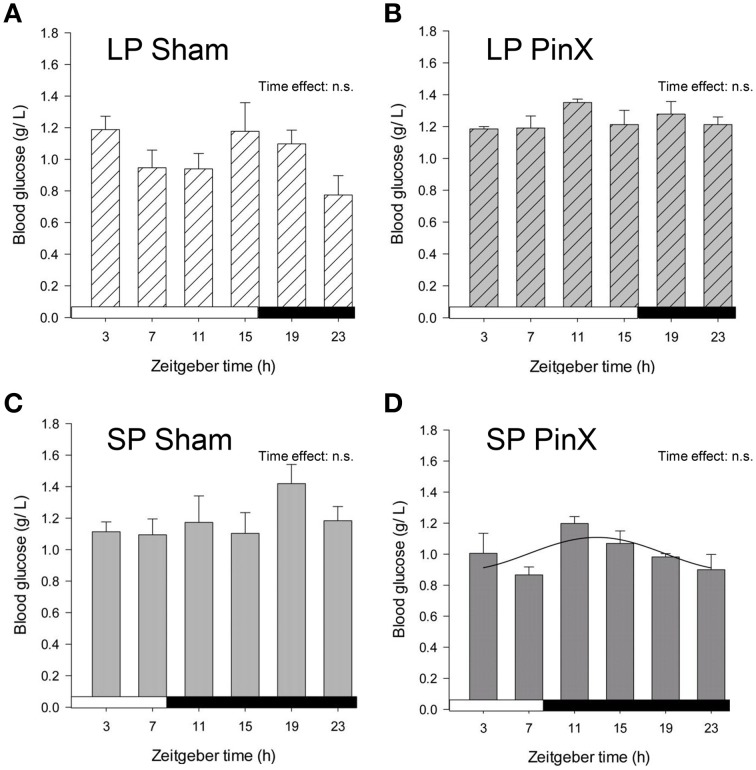
**Effects of photoperiod and pinealectomy on daily variations of plasma glucose in the** ***Syrian hamster*****.** Light and dark bars represent photoperiod and scotoperiod, respectively. LP, long photoperiod (16 h light/08 h dark) **(A,B)**; SP, short photoperiod (08 h light/16 h dark) **(C,D)**; Sham, sham-operated hamsters **(A,C)**; PinX, pinealectomized hamsters **(B,D)**. The absence of curves indicates that the daily variations of plasma glucose are not significant, except for SP PinX animals in which cosinor analysis detected a significant daily rhythm (for details, see Materials and Methods and Table 2). The inset for each graph indicates the effect of Zeitgeber time, evaluated by one-way ANOVAs or ANOVAs on ranks when normality test (Shapiro-Wilk) failed; n.s., non-significant. For a given panel, histograms lacking common letters are significantly different (*P* < 0.05).

Levels of blood glucose were hardly affected by Zeitgeber time (Three-Way ANOVA; *P* = 0.056), and unchanged by photoperiod (Three-Way ANOVA; *P* = 0.4) or treatment (Three-Way ANOVA; *P* = 0.6). The lack of daily rhythmicity in blood glucose might be due to the 4-h sampling. Nonetheless, the double interaction [Zeitgeber time × treatment] was highly significant (Three-Way ANOVA; *P* < 0.001). In particular, mean blood glucose in pinealectomized hamsters was significantly increased under long photoperiod, while it was decreased in short photoperiod (Fisher's *post-hoc* test; *P* < 0.001, Figure 4; Table 2). Moreover, mean glycemia in sham-operated hamsters was increased in short vs. long photoperiods (Fisher's *post-hoc* test; *P* = 0.007), while the values in pinealectomized animals was decreased in short vs. long photoperiods (Fisher's *post-hoc* test; *P* < 0.001). Cosinor analysis detected a significant daily rhythm of blood glucose only in pineactomized hamsters under short days (Figure 4; Table 2), precluding any comparison with the other studied groups. Nevertheless, these results indicate that circulating melatonin differentially affects overall glucose regulation according the photoperiod-induced metabolic changes.

## Discussion

The main findings of this study are that pineal melatonin not only acts as a time-giver for hormonal rhythms, but also affects glucose homeostasis in a photoperiod-dependent way.

**Figure 5:**
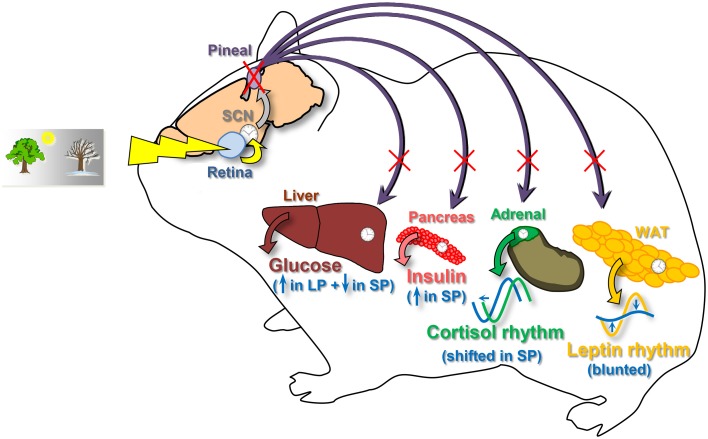
**Schematic view summarizing the effects of pinealectomy on metabolic physiology in the** ***Syrian hamster*****.** For each parameter, changes after pinealectomy are drawn in blue. Pinealectomy in *Syrian hamsters* suppresses the daily rhythmicity of plasma leptin synthesized by the white adipose tissue (WAT), whatever the photoperiod. Pinealectomy leads to shifts of the daily rhythm of plasma cortisol in hamsters adapted to a short photoperiod. Furthemore, pinealectomy leads to increased levels of insulin in hamsters exposed to a short photoperiod (SP). Finally, pinealectomy increases plasma glucose in leaner hamsters exposed to a long photoperiod (LP), and decreases glycemia in fattier animals adapted to a short photoperiod (SP). SCN, suprachiasmatic nuclei, site of the master clock.

The first conclusion is supported by the fact that pinealectomy in *Syrian hamsters* suppresses the daily rhythmicity of plasma leptin and can lead to shifts of the daily rhythm of plasma cortisol. The second conclusion relies on the differential impact of pinealectomy on glycemia and plasma insulin according to the photoperiod (Figure 5).

### Pinealectomy affects glucose and insulin levels according to photoperiods

This study is mainly based on cosinor analysis with a relatively small number of observations per time point (*n* = 4–6) and 4-h sampling (6 time-points over 24 h). In contrast to what is found in other rodents (La Fleur et al., 1999; Kalsbeek et al., 2001; Cuesta et al., 2009), no significant daily rhythm in blood glucose is found in control (sham-operated) *Syrian hamsters* housed under long or short photoperiods. This prevented us to assess the possible time-giving property of melatonin on that parameter. Based on a study in pinealectomized rats, the endogenous rhythm of pineal melatonin may enhance the amplitude of the daily rhythm of plasma glucose (la Fleur et al., 2001). In any case, the lack of rhythmic glycemia in *Syrian hamsters* has been already reported (Rowland, 1984). This specific feature is actually concomitant with a lack of marked day-night difference in feeding behavior (Rowland, 1984). Because hamsters in the present study were not fasted before blood sampling, meal-induced rise in plasma glucose may have blunted any small endogenous rhythm.

Contrary to mice fed *ad libitum* (Ahren, 2000), no daily variations of plasma insulin were found in intact *Syrian hamsters* exposed to either long or short photoperiods. Nonetheless, a significant rhythm of plasma insulin with low amplitude was only detected in pinealectomized hamsters. As noted for glucose levels, possible interactions with meal-induced secretion of insulin may have interfered with the endogenous rhythmicity of insulin. Alternativly, the fed state of the hamsters studied here avoided fasting-induced bias in hormonal/glucose levels due to variable mobilization of body stores according to photoperiods, times of day and associated differences in fasting duration.

Nevertheless, the present study provides novel information on how melatonin can modulate glucose homeostasis. We found that the effects of pinealectomy on plasma glucose and insulin depend on the metabolic state (i.e., short-day, fattier phenotype with more plasma glucose and leptin, and less insulin vs. long-day, leaner phenotype with less plasma glucose and leptin, and more insulin). In relatively lean hamsters (exposed to long photoperiod), pinealectomy leads to mild hyperglycemia. This finding is in accordance with the fact that pineal ablation in non-photoperiodic lean rats also increases nighttime glucose levels (la Fleur et al., 2001). This relative hyperglycemia at night in rats without pineal gland is not due to a reduced insulin secretion to meal cues because in hamsters exposed to long photoperiod, mean insulin levels were not significantly affected by pinealectomy. Accordingly, another work in pinealectomized rats did not detect changes in plasma insulin levels and provides experimental arguments for a decreased sensitivity of cells to circulating insulin (Alonso-Vale et al., 2004). Therefore, the mild hyperglycemia in pinealectomized hamsters in long days may be, as observed in rats, due to a reduced responsiveness of the target cells to insulin.

In sharp contrast, pinealectomy in relatively fatty hamsters (exposed to short photoperiod) reduces the mild hyperglycemia observed in sham-operated hamsters. Thus, pinealectomy in that case normalizes glycemia to levels close to those in long-day control hamsters. High-fat feeding triggers obesity in non-photoperiodic rats. Pineal ablation, however, does not modulate their concentration of blood glucose, at least in the morning (Prunet-Marcassus et al., 2003). In the present work, we also found that concentrations of plasma insulin were increased by pinealectomy in hamsters exposed to short photoperiod. Therefore, considering that insulin sensitivity is improved under short days compared to long days, the increased concentrations of circulating insulin can explain the lower levels of blood glucose. Meanwhile, our results reveal that the lack of circulating melatonin in hamsters markedly modifies glucose homeostasis, with opposite effects according to their seasonal metabolic state.

### Pinealectomy shifts the daily rhythm of cortisol

In sham-operated hamsters, the daily pattern of plasma cortisol was found to be unimodal and bimodal in short and long photoperiods, respectively. Furthermore, plasma levels of cortisol are larger in short compared to long photoperiods. This contrasts with previous results in the Syrian hamster that found unchanged levels in males (de Souza and Meier, 1987) or decreased levels of cortisol in short days (Ottenweller et al., 1987; Nexon et al., 2011). These discrepancies are puzzling, and may be related to the more extreme photoperiods used in the present study and the fact the hamsters here were all operated upon. Furthermore, the reported quiescent hypothalamo-hypophyso-adrenal axis in *Syrian hamsters* under short photoperiods can be profoundly modulated by social interactions (Morgan, 2012). It is therefore possible that the present housing conditions (3–5 animals per cage) may have led to social instability and/or inter-individual differences in social status, thus keeping an activated hypothalamo-hypophyso-adrenal axis in *Syrian hamsters* under short photoperiods. Regarding the effects of melatonin on glucorticoids, pinealectomy in *Syrian hamsters* does not affect the mean levels of cortisol, whatever the photoperiod. This confirms what is found by several studies in rats for corticoterone levels (Szafarczyk et al., 1983; Kaplanski and Ronen, 1986), albeit other investigations report increased levels of plasma corticosterone in pinealectomized rats (Oxenkrug et al., 1984; Alonso-Vale et al., 2004).

In terms of daily timing, a clear phase-advance is induced by pinealectomy in hamsters exposed to short days. Previous studies in adult rats found no effect of pinealectomy on the phase of corticosterone profile (Szafarczyk et al., 1983; Kaplanski and Ronen, 1986), while adrenal glands of rat fetus is entrained by maternal melatonin (Torres-Farfan et al., 2011). Furthermore, melatonin modulates *in vitro* molecular oscillations in the adrenal gland of adult monkeys (Valenzuela et al., 2008). Together, these findings suggest that circulating melatonin can participate in the control of adrenal rhythmicity.

### Pinealectomy flattens the daily rhythm of leptin

In accordance with the increased adiposity in hamsters exposed to short photoperiods (Bartness and Wade, 1985), mean levels of plasma leptin are higher in hamsters exposed to short photoperiods in comparison with animals in long photoperiods, as found by others (Horton et al., 2000). Furthermore, whatever the photoperiod, pinealectomy in hamsters does not affect mean leptin levels, in keeping with results in the non-photoperiodic laboratory rat (Alonso-Vale et al., 2004).

In the present study, the peak of plasma leptin in hamsters exposed to long or short photoperiods takes place at night, as in other nocturnal rodents like rats and mice (Ahren, 2000; Kalsbeek et al., 2001; Cuesta et al., 2009). Other reports in *Syrian hamsters*, however, found either hardly rhythmic profile (Horton et al., 2000) or rhythmic leptin peaking during daytime (Karakas and Gunduz, 2006). The daily rhythm of leptin is thought to be controlled both by the master clock in the SCN and by the adipose clock (Kalsbeek et al., 2001; Karakas and Gunduz, 2006; Otway et al., 2009). In addition, in cultured adipocytes, rhythmic treatment with melatonin has been shown to synchronize their metabolic and hormonal function, including leptin secretion (Alonso-Vale et al., 2008). This result perfectly fits with our *in vivo* demonstration that in hamsters without circulating melatonin at night, the daily rhythm of leptin is blunted. These findings highlight that rhythmic melatonin is a time-giver for rhythmic secretion of leptin. To firmly demonstrate that interpretation, further experiments in pinealectomized hamsters treated with melatonin are needed. It is expected that in contrast to continuous melatonin replacement, only restoration of a melatonin rhythm will reinstate daily variations of plasma leptin.

### Organ-specific sensitivity to rhythmic melatonin or glucocorticoids

Because of the presence of melatonin receptors in a multitude of central and peripheral organs, the daily rhythm of plasma melatonin is thought to distribute temporal cues generated by the master clock throughout the body, thus playing a role of coupling between the central and secondary clocks (Pevet and Challet, 2011). In the present study, we noticed that pinealectomy affects daily rhythms of plasma leptin, cortisol, and insulin in different ways depending on the rhythm considered (Figure 5). We suggest that these differential effects of melatonin (or its absence) *in vivo* are indicative of direct effects on targeted peripheral tissues, rather than indirect effects on upstream structures (e.g., in the central nervous system). Previous studies on organs (white adipose tissue, adrenal gland or pancreas) isolated *in vitro* are in accordance with this hypothesis (Peschke and Peschke, 1998; Alonso-Vale et al., 2008; Valenzuela et al., 2008).

It is worth reminding that rhythmic glucocorticoids, such as cortisol and corticosterone, are also considered themselves as internal time-givers. The prevalent view is that most, if not all, peripheral organs that express glucocorticoid receptors are sensitive to the synchronizing effects of applications of glucocorticoid agonist (dexamethasone) (Balsalobre et al., 2000). Therefore, a given structure expressing the *ad hoc* receptors could theoretically be sensitive to the two hormonal synchronizers. Accordingly, in the white adipose tissue, both glucocorticoids and melatonin can modulate timing of daily profiles of metabolic gene expression (Alonso-Vale et al., 2008; Su et al., 2015), or hormonal output (i.e., leptin; Alonso-Vale et al., 2008; this study). In fact, this dual sensitivity may not be so widespread.

Some tissues, indeed, appear to be more specifically sensitive to the synchronizing effects of either melatonin or glucocorticoids. In the mouse liver for instance, glucocorticoids play a strong synchronizing effect, as shown by arrhythmicity in metabolic gene expression after adrenalectomy (Oishi et al., 2005), while melatonin cues do not markedly affect hepatic timing (Houdek et al., 2015). In the brain of mice, the daily variations of dopamine in the striatum are controlled by rhythmic melatonin, as evidenced by disappearance of rhythmicity after pineal ablation and restoration with daily injections of melatonin, while adrenalectomy does not impair the timing of the dopaminergic variations (Khaldy et al., 2002). Conversely, daily variations of serotonin in the rodent brain, known to be controlled by rhythmic glucocorticoids (Segall et al., 2006; Malek et al., 2007), are not affected by pinealectomy or daily injections of melatonin (Khaldy et al., 2002). Finally, circadian timing in the pars tuberalis of the hypophysis appears to be driven by circulating melatonin (Dardente, 2007).

These data, together with our differential findings on plasma cortisol and leptin after pinealectomy, favor the hypothesis that rhythmic melatonin acts as an internal synchronizer on targeted structures. The functional implication of these specific temporal regulations for physiology and health remains to be investigated. The seasonal species that display annual and reversible changes in metabolic physiology, like *Syrian hamsters* studied here, provide promising models in that respect.

### Conflict of interest statement

The authors declare that the research was conducted in the absence of any commercial or financial relationships that could be construed as a potential conflict of interest.
